# THE PHYSIOLOGIC COMPLEXITY OF BEAT-TO-BEAT BLOOD PRESSURE FLUCTUATION IS ASSOCIATED WITH FRAILTY IN OLDER ADULTS

**DOI:** 10.1093/geroni/igac059.1157

**Published:** 2022-12-20

**Authors:** Junhong Zhou, Yurun Cai, Yi Guo, Xin Jiang

**Affiliations:** Harvard Medical School/Hebrew SeniorLife, Roslindale, Massachusetts, United States; University of Pittsburgh, Pittsburgh, Pennsylvania, United States; Shenzhen People’s Hospital, Shenzhen, Guangdong, China (People's Republic); Shenzhen People’s Hospital, Shenzhen, Guangdong, China (People's Republic)

## Abstract

**Background:**

Beat-to-beat blood pressure (BP) is an important cardiovascular output and regulated by neurophysiologic elements over multiple temporal scales. The multiscale dynamics of beat-to-beat BP fluctuation has thus been characterized using “BP complexity” and has been linked to age-related adverse health outcomes. We here aimed to examine the relationship of BP complexity to frailty, and if BP complexity mediates the association between arterial stiffness and frailty. Method: A total of 350 older adults completed assessments for frailty (i.e., Fried frailty phenotype criteria), arterial stiffness (i.e., average brachial-ankle pulse wave velocity), and beat-to-beat finger BP. The complexity of beat-to-beat systolic (SBP) and diastolic (DBP) BP series were measured using multiscale entropy. The relationships between frailty, BP complexity and arterial stiffness were examined using ANOVA and linear regression models. The effects of BP complexity on the association between arterial stiffness and frailty were examined using mediation analyses.

**Results:**

Compared to non-frail, pre-frail and frail groups had significantly elevated lower SBP and DBP complexity (F>11, p< 0.001), and greater arterial stiffness (F=16, p< 0.001). Greater arterial stiffness was associated with lower BP complexity (β<-0.42, p< 0.001). SBP and DBP complexity mediated the association between arterial stiffness and frailty (indirect effects>0.28), account for at least 47% of its total effects on frailty (Mediated proportion: SBP: 50%, DBP: 47%).

**Conclusion:**

This study demonstrates that BP complexity is associated with frailty in older adults, and mediates the association between arterial stiffness and frailty, suggesting that this metric would serve as a marker to characterize important functions in older adults.

